# Effects and Long-Term Outcomes of a Modified Triple-P Procedure in Patients With Severe PAS: A Retrospective Cohort Study

**DOI:** 10.3389/fmed.2022.839716

**Published:** 2022-03-30

**Authors:** Huidan Zhao, Xianlan Zhao, Chen Chen, Ya Tao, Ruixia Guo

**Affiliations:** ^1^Department of Obstetrics and Gynecology, The First Affiliated Hospital of Zhengzhou University, Zhengzhou, China; ^2^Obstetric Emergency and Critical Care Medicine of Henan Province, Zhengzhou, China

**Keywords:** placenta accreta spectrum (PAS), abdominal aorta, balloon occlusion, peripartum hysterectomy, Triple-P procedure, conservative treatment/methods

## Abstract

**Background:**

The distinguished Triple-P procedure has been reported as a conservative surgical alternative to peripartum hysterectomy for placental accreta spectrum (PAS). In this study, we modified the procedure combined with prophylactic abdominal aorta balloon occlusion and/or tourniquet and evaluated the effect and long-term outcomes.

**Methods:**

This was a retrospective study involving pregnant patients with clinically confirmed severe PAS (including placenta increta and percreta) between January 1st, 2017 and June 30th, 2020 in the First Affiliated Hospital of Zhengzhou University. A total of 334 pregnant women were recruited in this study. The 142 women that were subjected to modified Triple P Procedure were regarded as the observation group while 194 pregnant women that were treated with other sutures were regarded as the control group. Demographic characteristics, placental accreta spectrum score (PAS score), estimated blood loss (EBL), operative time, blood transfusion rate and volume, neonatal weight, post-operative hospital stays and costs were evaluated. Short-term complications, including fever, hematoma, thrombus, bladder rupture and intensive care unit (ICU) transfer rate, as well as long-term outcomes including breast feeding, menstruation, intrauterine adhesion, and chronic abdominal pain among others were followed up in the outpatient clinic and by phone calls.

**Results:**

For all cases, EBL was lower in the observation group than in the control group, 1,200 (687–1,812) ml and 1,300 (800–2,500) ml, respectively. The difference was statistically significant (*P* < 0.05). Operative time were statistically significantly shorter in the observation group [99.5 (84.0–120.0) min and 109.0 (83.8–143.0) min, *P* < 0.05]. Lengths of postoperative hospital stays were 4 (4–7) and 5 (4–7) days in the observation and control group, which was significantly shorter in the observation group (*P* < 0.05). There were no significant differences in PAS scores, blood transfusion volume, neonatal weight, fever, hematoma, thrombus, bladder rupture and ICU transfer rates between the two groups. All patients, except one in control group, had preserved uterus. There were no statistically significant differences in short-term and long-term complications between two groups.

**Conclusion:**

In summary, when combined with tourniquet and/or prophylactic abdominal aorta balloon occlusion, modified Triple-P procedure may be effective in reducing intraoperative blood loss and hysterectomy in patients with placenta increta/percreta. It is a safe and effective surgical alternative to peripartum hysterectomy. However, the complications associated with interventional radiology service should be evaluated furthermore.

## Introduction

With implementation of the 3-child policy in China, an increasing number of women with advanced maternal ages are choosing to have another child. A continuous increase in cesarean delivery rates has resulted in a substantial rise in women with prior cesarean delivery scars (1). Moreover, applications of assisted reproductive technologies have significantly increased. These risk factors contribute to placenta accreta spectrum (PAS) disorders. PAS is associated with a very high risk of obstetric and pediatric complications, especially postpartum hemorrhage (PPH), which often leads to secondary complications including shock, coagulopathy, disseminated intravascular coagulation (DIC), multi-organ failure (MOF), and even death (1, 2).

Traditionally, a peripartum hysterectomy has been preferred for the treatment of PAS (1, 2). Hysterectomy is an effective method on arresting severe postpartum hemorrhage. But it causes permanent loss of fertility and brings physiological and psychological problems to pregnant women (3, 4). Conservative surgical management options have been reported, including distinguished Triple-P procedure, proposed by Chandraharan et al. (5). Various uterus compression sutures for clinical management of placental previa accreta have been reported, each with varying advantages and complications (5–11).

Based on the invasive depth of the trophoblast, three subtypes have been differentiated: placenta creta, placenta increta, and placenta percreta (1, 12). Placenta increta and percreta is severe subtype of PAS and easily leads to severe maternal and fetal complications. Our procedure modified the Triple-P procedure about the second “P,” pelvic devascularization, and hemostasis method after the placental tissue removed. In our procedure, one occlusive balloon is placed in the abdominal aorta artery to achieve devascularization instead of two occlusive balloons placed in the bilateral internal iliac artery in the Triple-P procedure. For most of patients, tourniquet were used simultaneously, which can achieve further blood occlusion. As for the hemostasis method after the placental tissue removed, a single continuous suture was performed on the lower lip of the uterine incision before the incision was closed, which was one part of placental percreta area and was full of tortuous vessels. This step can significantly reduce the bleeding. This strategy focuses on bleeding of the anterior myometrium, which is the most common and serious part of placenta that is affected. We evaluate the effects and long-term follow-up outcomes of this method.

## Materials and Methods

### Participants

A total of 925 pregnant women with singleton and placental accreta spectrum delivered by cesarean section at the first affiliated Hospital of Zhengzhou University from January 2017 to June 2020. There were 336 cases of placental increta/percreta. A total of 142 pregnant women without severe abdominal adhesion and most of the placenta was implanted in the anterior wall of uterus. They were suitable for modified “Triple-P” procedure and were subjected to observation group, while 194 pregnant women who were not suitable for modified triple P procedure were assigned to control group. Many other procedures were performed depending on the situation and the evaluation before surgery after manual removal of the placenta. These suture methods included figure of eight suture, coarctation suture, and simple continuous suture among others. To the patients with deep cervical or vaginal invasion, radial modified figure of eight suture around cervix proposed by our team was performed which was published on Chinese Journal of Obstetrics and Gynecology (13). Interventional embolization was necessary occasionally.

Before surgery, we performed ultrasonic and Magnetic Resonance Imaging (MRI) examination to all PAS suspected patients. Data about the placenta, including its localization, thickness, loss of hypoechoic retroplacental zone, utero-vesical hypervascularity, placental neovascularization pattern and numerous large and irregular lacunae in the placenta, abnormal uterine bulging, dark intraplacental bands on T2-weighted images, heterogeneous signal intensity and disorganized placental vasculature were recorded.

**Figure 1:**
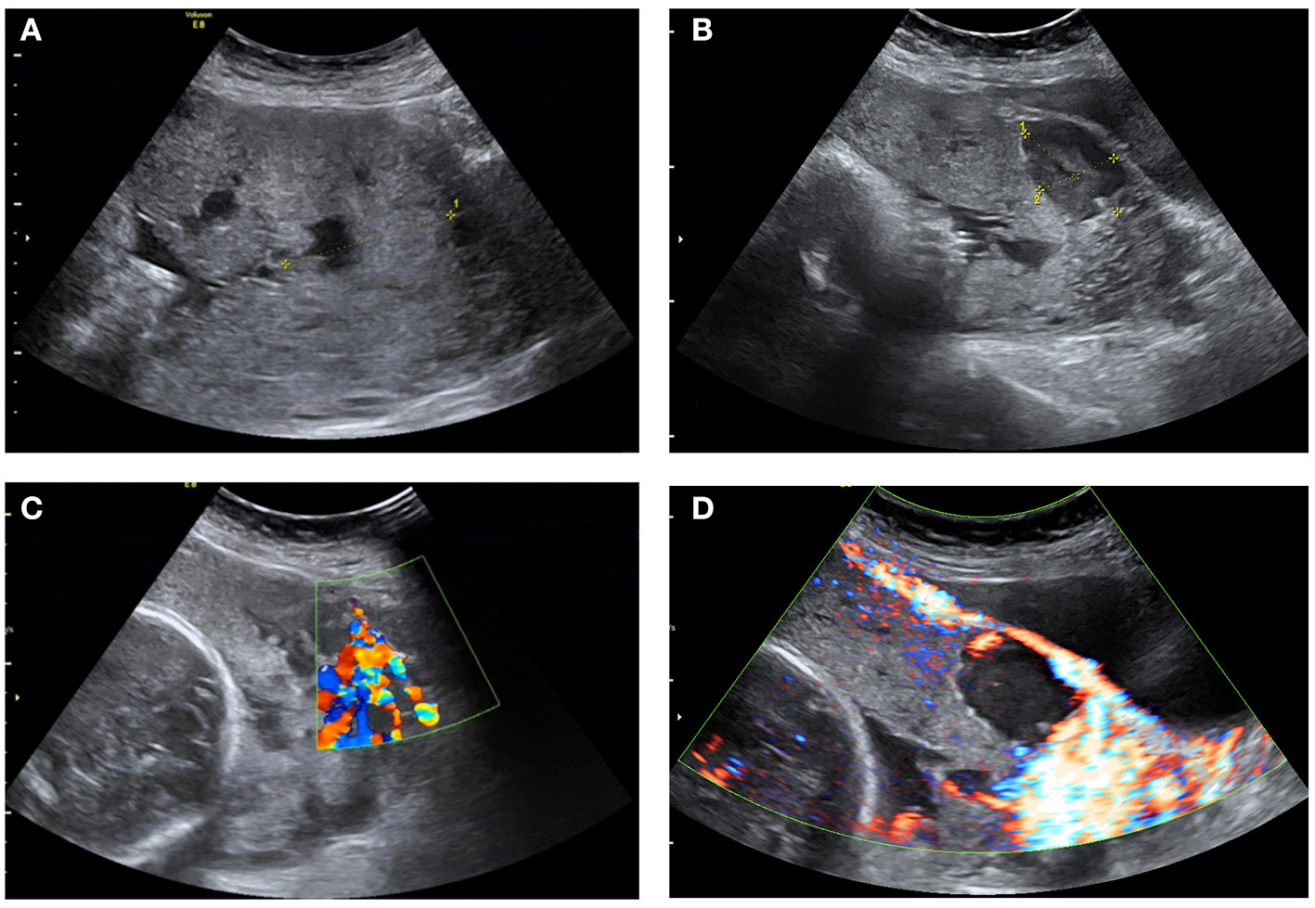
Ultrasonograms of the participant. **(A)** Increased placental thickness under preoperative ultrasound examination; **(B)** Loss of hypoechoic retroplacental zone and numerous large and irregular lacunae in the placenta; **(C)** Absence of the lower muscle layer of uterus and Vessels with high velocity blood flow leading from the myometrium into the placental lacunae; **(D)** Striking amount of color Doppler signal seen between the myometrium and the posterior wall of the bladder.

**Figure 2:**
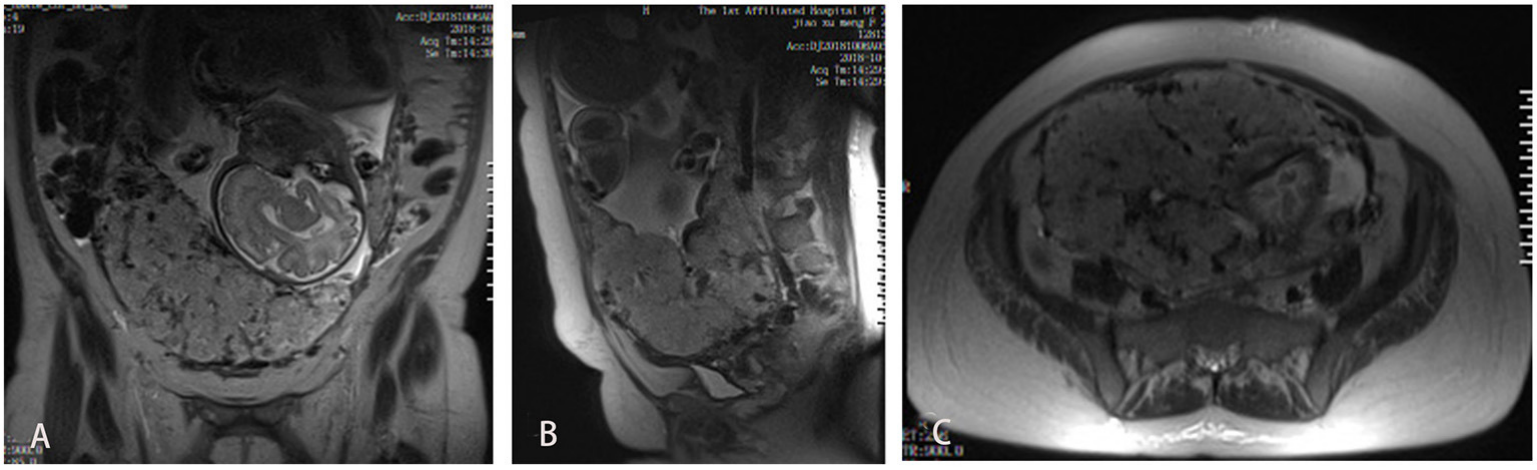
MRI images of the participant. **(A)** MRI coronal plane on T2-weighted images, showing thickened placenta, heterogeneous signal intensity; **(B)** MRI sagittal plane, showing unclear boundary of placenta and uterine wall; **(C)** MRI cross section, showing focal interruption of myometrium.

Inclusion criteria: All the patients were confirmed by ultrasound and MRI as well as clinical diagnosis. (i) PAS score under ultrasound ≥ 8. PAS score refers to the scoring system designed by Peking University Third Hospital (14) (Figure 1); (ii) Placenta increta/percreta confirmed by MRI (2) (Figure 2); (iii) Surgical manifestations (FIGO grade 2 and 3) (12), such as invasion of placental villi into the uterine muscular fibers, uterine serosa, urinary bladder or broad ligament, vaginal wall, pelvic sidewall or any other pelvic organ. Pregnant women who met all the above criteria were diagnosed with placental increta/percreta. The exclusion criteria were: (i) PAS score under ultrasound <8; (ii) Patients with severe obstetric complications and serious internal and surgical diseases, including cardiac disease, liver disease or pre-eclampsia; (iii) Patients with twins or multiple pregnancy.

All surgical procedures were performed by the same surgeon, Xianlan Zhao. The whole process was managed by our multidisciplinary team, including doctors who were experts in ultrasound, MRI, anesthesiology, invasive technology and obstetrics. At 42 days after surgery, patients returned to the outpatient clinic of the hospital. Clinical follow-ups were made by phone calls at 6 months, 1 year, and 2 years after surgery.

### Surgical Procedures

**Figure 3:**
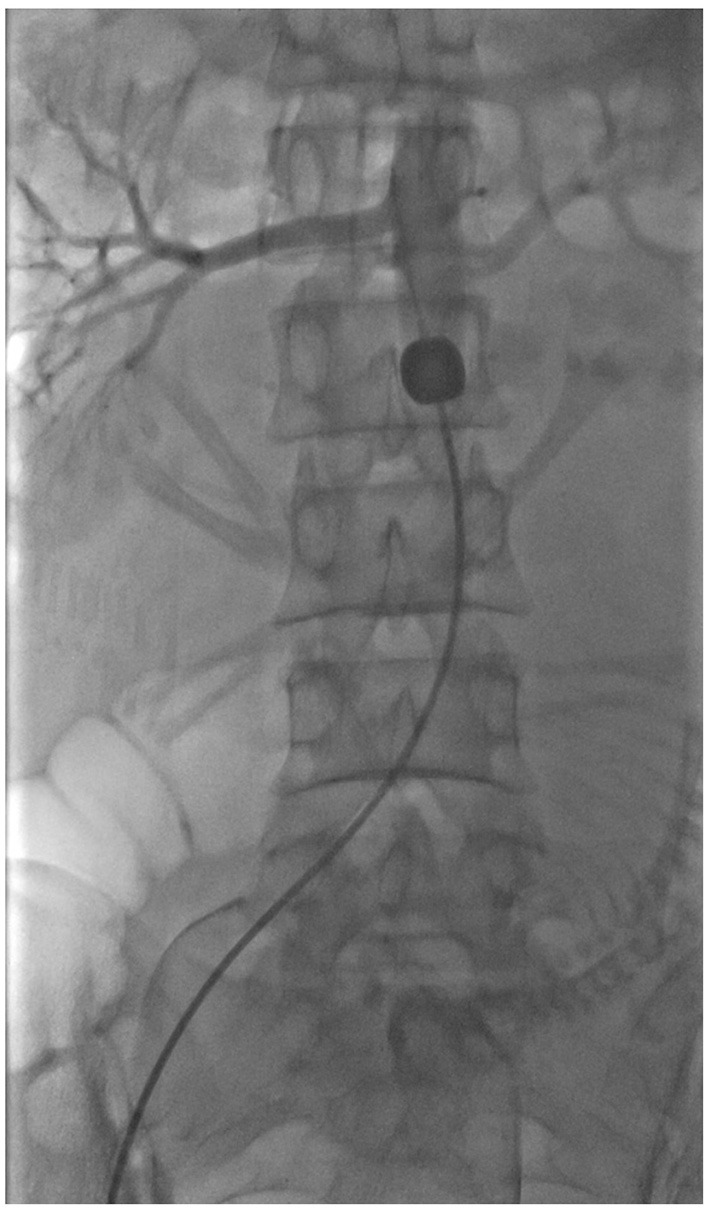
Image of the balloon. The balloon was placed into the distal abdominal aorta beneath the opening of the renal arteries.

**Figure 4:**
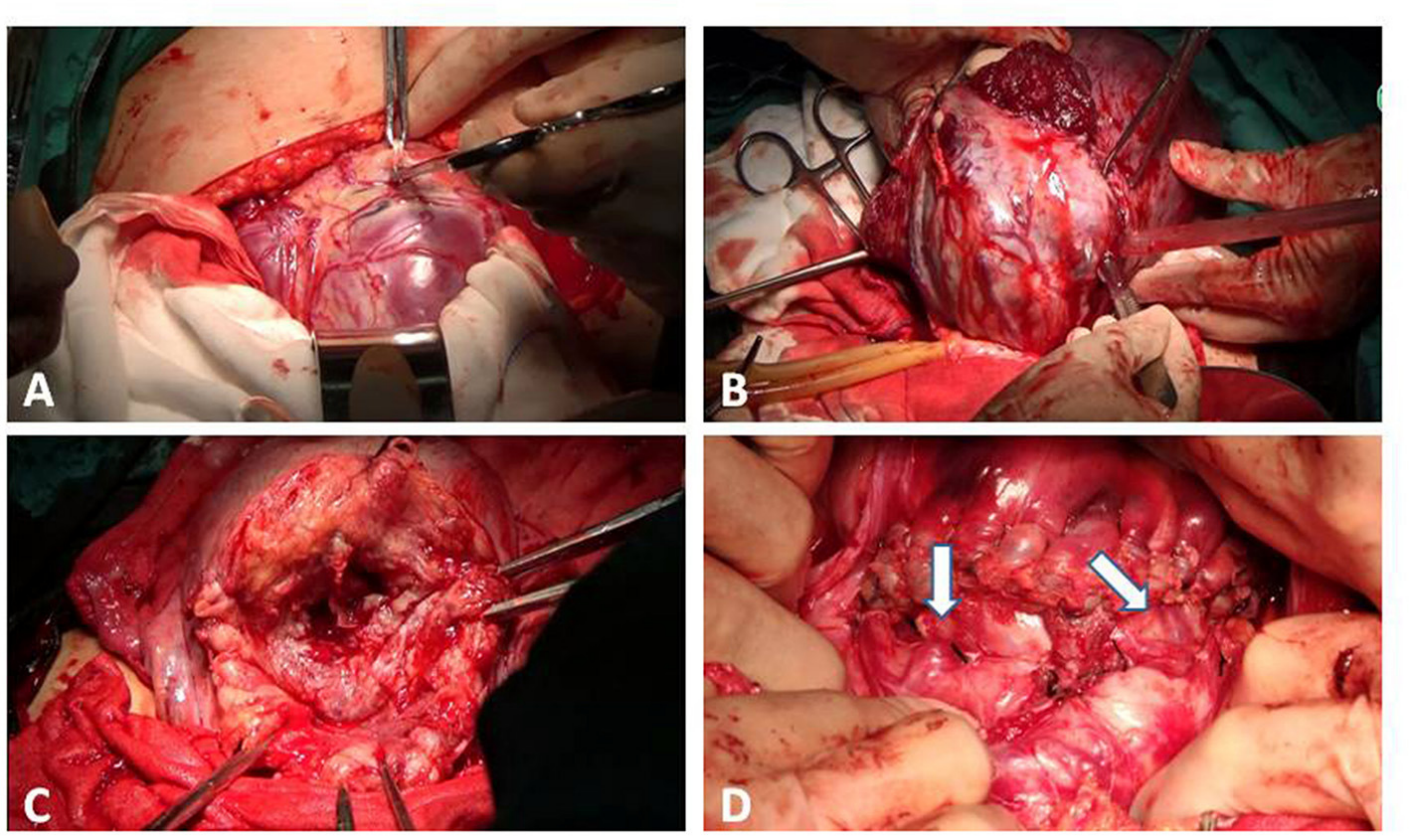
Process of the procedure. **(A)** The uterine incision was over the upper border of the placenta. **(B)** A tourniquet was put as low as possible at the lower segment of the uterus. The bladder was pushed down and the anterior myometrium was partially resected with the placenta unseparated. **(C)** A single continuous suture was made along the lower lip of the uterine incision. **(D)** The lower transverse incision at the uterine was sutured in one layer. This area was still permeated with tortuous vessels (the arrow).

Individual schedules were made for every patient, including gestational week to pregnancy termination, amount of prepared blood, and necessity for placing a prophylactic abdominal aorta balloon among others. For the following three types of situations, the abdominal aorta balloon was preset before surgery: (i) Patients with more than one previous cesarean section; (ii) Severe abdominal adhesion in previous cesarean section predicted the difficulty of tourniquet placement during this operation; (iii) Patients with cervical involvement as indicated by ultrasound or MRI. Surgical procedures were performed in the Digital subtraction angiography (DSA) room. Before cesarean section, the interventional doctor inserted an 8F balloon catheter into the distal abdominal aorta beneath the opening of the renal arteries through the right femoral artery. A new compliant 14 mm × 10 mm Fogarty balloon catheter (Edwards Lifesciences, USA) was used. After confirming the good position of the balloon, cesarean section was performed, Figure 3.

i. The primary incision was recut. Majority of the cases were transverse incisions of the lower abdomen. Our incision was about 3 cm longer than the previous one to achieve better exposure.

ii. Uterine incision was performed transversely over the upper border of the placenta, which is obvious after opening the abdomen. As shown in Figure 4A, there were engorged and tortuous vessels on the serosa surface corresponding to the area of the placental increta beneath.

iii. After delivering the fetus, the uterus was immediately exteriorized out of abdominal cavity. Adhesion between uterus and abdominal wall should be separated.

iv. At the same time, the interventional doctor injected 1.2–1.6 ml of saline into the balloon to block the abdominal aorta. Effective occlusion was achieved when the pulse oximeter decreased to 0 as assessed by distal foot–toe monitor. The single blocking time of the aortic balloon was 15 min with a 1-min interval. This procedure could be performed repeatedly. Total time did not exceed 45 min. Meanwhile, we put a tourniquet as low as possible at the lower segment of the uterus to further block blood flow.

v. Bladder was pushed down to a horizontal line of internal cervix os or to the accreta area if the posterior wall of the bladder muscle layer was invaded. The anterior myometrial from the lower lip of the uterine incision to the area about 2 cm above the cervix was resected using a scalpel or scissor together with the bulk of implanted placenta (Figure 4B).

vi. A single continuous suture was made along the lower lip of the uterine incision, as shown in Figure 4C. Other hemostatic methods were performed around the internal cervix os and the area where the placenta could not be excised. The figure-of-eight suture was the first choice for us. Based on hemostasis, the saline in the balloon was gradually extracted until the balloon was completely deflated. The tourniquet was removed.

vii. The lower transverse incision at the uterine was sutured in one layer in the common way, or two layers if necessary. As shown in Figure 4D, this area was still permeated with tortuous vessels. In cases in which suturing the second layer was considered dangerous, we preferred monolayer suturing.

viii. The balloon catheter was removed after surgery. When necessary, uterine arterial ligation was performed on patients with consistent vaginal bleeding (see Supplementary Video 1).

Demographic characteristics including age, gravidity, time of previous c-sections, gestational age and PAS score were recorded. We also recorded the perioperative information of patients, including estimated blood loss (EBL), operative time, blood transfusion type and volume, neonatal weight, postoperative hospital stay period, costs and whether they had uterine arterial embolism (UAE) and intensive care unit (ICU) transfer. Autologous blood transfusion technology was performed for all study participants based on their wishes. Four parts were included in EBL: amount of blood in negative pressure suction bottle, amount of blood in autologous retrieved device, increased weight of dressing and amount of vaginal hematocele cleared at the end of the operation.

Short-term follow-ups for all patients were performed at the inpatient department and the outpatient department 42 days after the operation. Short-term complications included bladder injury, fever (temperature > 37.3°C), late puerperal hemorrhage, pyometra, peritonitis, uterine necrosis, sepsis and perioperative infection, hematoma around the puncture location, false aneurysm, arteriovenous fistula and thrombus. Patients with an abdominal aorta arterial balloon were subjected to an echo examination after surgery to determine whether there was thrombus in the vessels of the pelvis and extremities.

Long-term follow ups were done by phone. Long-term follow-up was performed at 6 months, 1 year, and at 2 years after surgery. Breast feeding situation, menstrual quantity, menstrual period, chronic pelvic pain, intrauterine adhesion, and lower extremity discomfort were assessed.

### Statistical Analysis

The SPSS 26.0 software was used for data analysis. Age, number of pregnancies, previous cesarean section times, gestational weeks, PAS score, cost, operation time, EBL, CRBC transfusion, autologous blood transfusion, postoperative hospital stay and neonatal weights were measurement data. Age, cost and neonatal weights were normally distributed and were presented by mean ± standard deviation ($\bar{x}\;\pm \;s$). The independent sample *t*-test was used for inter group comparisons. Data for the other indicators did not conform to normal distribution, and were presented by median (25th−75th percentile) [M (p25–p75)]. The rank sum test was used for inter group comparisons. The remaining statistical indicators were count data, expressed by frequency or percentage, and the chi square test was used for inter group comparisons. *p* ≤ 0.05 was set as the threshold for statistical significance.

## Results

### General Characteristics of Patients

All the 336 participants had complete placenta previa. The characteristics of the participants are shown in Table 1. Mean ages of the observation group and the control group were 32.6 ± 4.4 years, 32.0 ± 5.0 years. The median number of gestational times for both groups was 4 while the number of previous C-sections for both groups was 1. The median gestational age of delivery for both groups was 35 weeks. The median PAS scores for the observation group and the control group were 10.5 and 11. The above outcomes were not statistically significant between the two groups. Emergency C-section was performed because of prenatal vaginal bleeding in 11 cases (6 in the observation group and 5 in the control group). The rest of the 325 cases were planned procedures. There were 19 patients with cervical invasion in the observation group and 28 cases in the control group. Placental villi were seen to be invading into the bladder with 8 patients of the observation group and 11 cases of the control group. Broad ligament was found invaded with 5 patients (2 in the observation group and 3 in the control group.) All the differences were not statistically significant.

**Table 1:** Comparation of demographic characteristics of the patients with placenta increta/percreta in two groups.

**Group**	***n***	**Age (years,** **$\bar{x}\;\pm \;s$ ±*s*)**	**Gravidity [M (P_**25**_-P_**75**_)]**	**Previous c-sections [M (P_**25**_-P_**75**_)]**	**Gestational age [wk, M (P_**25**_-P_**75**_)]**	PAS score*[M (P_**25**_-P_**75**_)]	**Emergency c- section [*n* (%)]**
Modified Triple-P	142	32.6 ± 4.4	4 (3–5)	1 (1–2)	35 (34.1–35.9)	10.5 (9.0–12.0)	6 (4.2)
Control	194	32.0 ± 5.0	4 (3–5)	1 (1–2)	35 (33.4–35.8)	11.0 (9.8–12.0)	5 (2.6)
Statistic value		1.06	1.08	0.72	0.66	0.26	0.70
*P*-value		0.292	0.19	0.47	0.51	0.79	0.40

* *PAS score refers to the scoring system designed by Peking University Third Hospital*.

### Comparison of Perinatal Outcomes of Two Groups

Perioperative data of patients is shown in Table 2. Procedures were performed under tourniquet in 108 cases, and under prophylactic abdominal aorta balloon occlusion (PABO) in 104 cases of the observation group. Seventy-five cases were treated using both strategies, while 5 cases were treated using neither of the methods. In the control group, the procedures were performed under tourniquet in 138 cases, and under PABO in 136 cases. Ninety-nine cases were treated using both strategies, while 19 cases were treated by neither of the methods. The EBL was lower in the observation group than in the control groups, 1,200 (687–1,812) ml and 1,300 (800–2,500) ml, respectively. Operative time and the lengths of postoperative hospital stays were significantly shorter in the bservation group than in the control groups [Operative time, 99.5 (84.0–120.0) min and 109.0 (83.8–143.0) min; postoperative hospital stays, 4 (4–7) and 5 (4–7.5) days, respectively]. The observation group exhibited significantly better outcomes than the control group. There were 78 cases in the control group and 53 cases in the observation group who chose autologous blood transfusion technology. The volumes of transfusion were 581 (272–785) ml and 415 (328–662) ml, respectively. Moreover, there were 122 cases in the control group and 77 cases in the observation group who were subjected to allogeneic blood transfusion with the volume of transfusion being 415 (332–655) and 555 (267–785) ml, respectively, which was less in the observation group than in the control group, but the difference was not significant. The procedural costs for the observation group and control groups were 28,486 ± 8,574 and 32,384 ± 11,689 RMB, respectively. The cost was significantly lower in the observation group than in the control group.

**Table 2:** Comparation of perioperative data of the patients with placenta increta/percreta treated by two different procedures.

**Group**	**Costs [RMB, ($\bar{x}\;\pm \;s$ ±s)]**	**Tourniquet [*n* (%)]**	**PABO [*n* (%)]**	**Operative time [min, M (P_**25**_-P_**75**_)]**	**Estimated blood loss [ml, M (P_**25**_-P_**75**_)]**	**CRBC transfusion [ml, M (P_**25**_-P_**75**_)]**	**Autologous blood transfusion [ml, M (P_**25**_-P_**75**_)]**	**Length of postoperative hospital stay [days, M (P_**25**_-P_**75**_)]**	**Neonatal weight [g, $\bar{x}\;\pm \;s$ ±s]**
Modified Triple-P	28,486 ± 8,574	108 (76.1)	104 (73.2)	99.5 (84.0–120.0)	1,200 (687–1,812)	400 (0–800)	415 (332–655)	4 (4–7)	2,652 ± 614
Control	32,384 ± 11,689	138 (71.1)	136 (70.1)	109.0 (83.8–143.0)	1,300 (800–2,500)	800 (0–1,200)	555 (267–785)	5 (4–7)	2,671 ± 649
Statistic value	3.37	1.01	0.40	2.09	2.06	1.41	1.60	3.31	0.26
*P*-value	0.001*	0.31	0.53	0.04*	0.04*	0.16	0.11	0.01*	0.80

* *P < 0.05. PABO, abdominal aorta balloon occlusion; CRBC, concentrated red blood cells*.

Postoperative data of the patients are shown in Table 3. There were 6 cases (2 in the observation group and 4 in the control group) whose uterine arteries were embolized to stop bleeding. However, all uteruses, except one in the control group, were successfully preserved. Bladder injury occurred in 4 patients of the control group and in 3 cases of the observation group. The injury happened when pushing down the bladder and while separating the reflection between the bladder and uterus. One patient that used a urinary catheter for 18 days to improve bladder healing was confirmed with a urinary fungal infection. In the other 6 patients with a urinary catheter, it remained for 3–14 days. Tube ligation was performed on 111 patients, based on their wishes and the risk of next pregnancy, while 31 patients refused peripartum sterilization. Six patients were sent to the ICU because of hemorrhagic shock or unstable vital signs. There was no maternal mortality in both groups.

**Table 3:** Comparation of postoperative follow-up data of the patients with placenta increta/percreta treated by two different procedures.

**Group**	**UAE [*n* (%)]**	**Fever [*n* (%)]**	**Hematoma [*n* (%)]**	**Thrombus [*n* (%)]**	**Bladder injury [*n* (%)]**	**Hysterectomy [*n* (%)]**	**ICU admission [*n* (%)]**
Modified Triple-P	2 (1.4)	12 (8.5)	4 (2.8)	4 (2.8)	3 (2.1)	0 (0)	2 (1.4)
Control	4 (2.1)	19 (9.9)	3 (1.2)	6 (3.1)	4 (2.1)	1 (0.5)	4 (2.1)
Statistic value	0.20	0.18	0.65	0.02	0.01	0.73	0.20
*P-*value	0.62	0.67	0.42	0.88	0.97	0.39	0.62

*UAE, uterine artery embolism*.

### Follow-Up

Follow-up findings from the study participants are shown in Tables 3, 4. There were no significant differences in all complications.

**Table 4:** Comparation of long-term follow-up data of the patients with placenta increta/percreta treated by two different procedures.

**Variables**	**Modified Triple-P ***n*** (%)**	**Control ***n*** (%)**	**χ^2^**	***P*** **-value**
Long-term follow-up			0.15	0.69
Succeeded	127 (89.4)	176 (90.7)		
Lost	15 (10.6)	18 (9.3)		
Time after the procedure				
More than 2 years	49 (34.5)	63 (32.5)	0.15	0.69
More than 1 year	113 (79.6)	148 (76.3)	0.51	0.48
More than 6 months	127 (89.4)	176 (90.7)	0.15	0.69
Breast feeding situation			0.29	0.59
Artificial feeding	14 (11.0)	23 (13.1)		
Breast feeding	113 (89.0)	153 (86.9)		
Menstruation restored			0.34	0.56
Yes	121 (95.3)	170 (96.6)		
No	6 (4.7)	6 (3.4)		
Time of menstruation resumed			0.79	0.67
<5 months	67 (52.8)	98 (55.7)		
5–10 months	42 (33.1)	50 (28.4)		
>10 months	18 (14.1)	28 (15.9)	.	
Menstrual quantity			0.67	0.71
Increase	5 (4.1)	6 (3.5)		
Same	104 (86.0)	142 (83.5)		
Decrease	12 (9.9)	22 (12.9)		
Menstrual period (days)			0.16	0.93
Longer	8 (6.6)	12 (7.0)		
Same	109 (90.1)	151 (88.8)		
Shorter	4 (2.7)	7 (4.1)		
Intrauterine adhesion	1 (0.8)	1 (0.6)	0.05	0.82
Light chronic pain of abdomen	5 (3.9)	4 (2.3)	0.71	0.40
Lower extremity discomfort	3 (2.4)	6 (3.4)	0.28	0.60

### Short-Term Complications

Thirty-one patients (12 in the observation group and 19 in the control group) developed a fever after surgery (37.3°C < axillary temperature). However, there were no cases that were complicated by pelvic abscess, purulent secretion or any other symptom of pelvic or uterine infection. Therefore, the fever was considered to be postoperative absorption heat or a moderate inflammatory reaction. Due to appropriate antibiotic administration, temperature recovered to normal before discharge.

Seven cases (4 in the observation group and 3 in the control group) had hematoma around the puncture location, 5 of which were small and disappeared before discharge. The hematoma was larger than 10 cm in the other 2 patients. Full recovery was confirmed in one of the two cases. The patient had a very low platelet count and she got extensive subcutaneous ecchymosis at the same time. The other patient had schizophrenia. She was lost to follow-up. Compression on the puncture location could not be effectively performed because of schizophrenia, which was probably the main reason for hematoma. Out of the 240 cases who placed aorta abdominalis, thrombus was found in 10 (4 cases in the observation group and 6 cases in the control group). Total incidence rate of thrombosis in all the subjects was 4.2%. Thrombus in seven cases (3 in the observation group and 4 in the control group) was in the artery around the puncture location, including external iliac artery, common femoral artery, superficial femoral artery and deep femoral artery, while 3 cases (1 in the observation group and 2 in the control group) were in the accompanying vein, including external iliac vein, common femoral vein, and superficial femoral vein, which could have been caused by compression in case of hemorrhages. Because of extensive external iliac artery and common femoral artery, one patient was treated by arteriotomy with embolectomy. Catheter-directed thrombolysis was provided for 3 patients while the other 6 cases were treated with thrombolysis by urokinase combined with LMWH anti-coagulation. These patients were followed up for 12–37 months, and they all had good prognoses, except one patient who felt numb in the right knee and ankle 30 months after surgery.

There were no cases of pyometra, peritonitis, uterine necrosis, sepsis or perioperative infection of any other system. Moreover, there was no late puerperal hemorrhage, false aneurysm, or arteriovenous fistula.

### Long-Term Complications

Up to December 31st 2020, 15 of the 142 patients in the observation group and 18 of the 194 patients in the control group were lost during follow-up while the other 127 patients in the observation group and 176 patients in the control group were successfully followed-up by phone for 3–38 months after surgery by the same doctor, Huidan Zhao. Long-term follow-up data of the patients are presented in Table 4.

Menstruation for 291 patients (170 in the observation group and 121 in the control group) resumed. In more than half of the patients, menstruation resumed within 5 months. After surgery, menstrual quantity decreased in 34 patients (22 in the control group and 12 in the observation group). Moreover, menstrual quantity reduced by more than half in 8 of the 34 cases, however, transvaginal ultrasonic examination for all the 34 patients was normal. Menstrual period shortened for 11 cases (7 in the control group and 4 in the observation group), at the same time, menstrual quantity decreased in all the 11 patients. Prolonged menstrual cycle was reported in 20 cases (12 in the control group and 8 in the observation group) and ranged from 3 to 12 days. Scar diverticulum was diagnosed in 6 cases by transvaginal ultrasound, while 14 patients were normal.

Nine patients exhibited light chronic pain in the lower abdomen and other uncomfortable symptoms, including 3 cases during intercourse and 1 case before urination.

Intrauterine adhesion was found in two patients. Surgical procedures for these two cases had been performed both under tourniquet and prophylactic abdominal aorta balloon occlusion. Adhesion was detected by transvaginal ultrasound and was diagnosed by hysteroscopy examination. Separation of intrauterine adhesions was performed under hysteroscopy.

Lower extremity discomfort was found in 9 patients (6 in the control group and 3 in the observation group). They had all been subjected to abdominal aorta balloon. The major symptoms were slight sense recession, numbness and soreness in the leg, knee or ankle, which arose from maintaining the same posture for a long time, staying in cold weather or exercising for a long period of time. One patient was diagnosed with thrombosis at the puncture location. Vessel radiography and ultrasonic examination were normal after treatment. The remaining 8 cases were confirmed by normal ultrasound performance during routine inspection 2 days after surgery.

After surgery, two women (1 in the control group and 1 in the observation group) with placenta percreta were able to get pregnant and deliver again. The placenta of the patient in the observation group was located at the posterior wall, far away from the internal os of the cervix. The placenta of the other case was located in the anterior wall of the uterus without placenta previa. Elective cesarean section with sterilization was successfully performed at full term gestation. Both of them exhibited good prognoses.

## Discussion

Maintaining the uterus under the safety premise is very important for PAS disorders. As the number of patients with PAS increase, many conservative management procedures have been developed to minimize uterine bleeding. These procedures include the extirpative technique (manual removal of the placenta), leaving the placenta *in situ*, one-step conservative surgery and the Triple-p procedure (1).

The “Triple-P procedure” proposed by British researchers Chandraharan E et al. involves perioperative placental localization, pelvic devascularization, placental non-separation with myometrial excision and reconstruction of the uterine wall, which effectively preserves the uterus (5, 15–17). The procedure has been applied and modified by other centers. The therapeutic effect has also been evaluated (18, 19). Our procedure modified the Triple-P procedure about the method of pelvic devascularization and hemostasis method after the placental tissue removed. Tourniquet plus one occlusive balloon instead of two internal iliac artery occlusive balloons leads to more effective devascularization of the uterus. A single continuous suture was performed on the lower lip of the uterine incision. Since the new-formed lower lip was one part of placenta implanted area that was full of tortuous vessels, which can cause massive bleeding in a short time, this single continuous suture significantly minimized the bleeding.

Based on the outcomes of our procedure and the success rate of conservative treatment in our study, the conservative method of uterine reservation with placenta separation is preferred for patients with stable vital signs, without rapid uncontrolled massive bleeding and without serious infections. Our method aimed at extensive placenta increta/percreta. Prophylactic devascularization achieved by aortic arterial balloon occlusion and/or tourniquet before surgery can reduce the risk of intraoperative blood loss and prevent the occurrence of severe postpartum hemorrhage (20–28).

We found that, compared to the control group, modified “Triple-P” procedure could significantly shorten the operation time, reduce perioperative blood loss, shorten postoperative hospital stays and reduced hospitalization costs. It reduced the amounts of autologous blood transfusion; however, the difference was not significant. There were no significant differences in postoperative complications such as UAE hemostasis, fever, thrombosis, puncture site hematoma, bladder injury, ICU admission and hysterectomy. First, the eroded defective anterior lower uterus segment and bulk of the placenta without detachment were removed, which reduced homeostasis area and shortened homeostasis time, consequently reducing bleeding volume. Second, once the partial anterior uterus myometrium had been resected, the internal os of the cervix was easier to expose, facilitating the next surgical homeostasis suture that was applied to this area. Third, only the eroded unfunctional uterus tissue was excised and the normal myometrium was preserved as much as possible. After reconstruction, the incision was the same as in a traditional C-section. Compared to other operations, this method has the advantages of less trauma.

Based on the standard of placenta accreta spectrum (1, 2), termination of pregnancy at 34–36 weeks for patients with regular perinatal care in our hospital was recommended. For some patients included in the study, it was after more than 36 weeks, because: (i) Irregular perinatal care in other hospitals, and gestational age at the time of referral being more than 36 weeks; (ii) After fully explaining the advantages and disadvantages of the method to patients, they paid too much attention to the complications of premature infants and refused to be operated before 36 weeks.

Long-term complications associated with conservative treatment for placenta increta/percreta have not been clearly elucidated. We have implemented this procedure for about 8 years. In our study, the effect of this procedure was evaluated. More importantly, the short-term and long-term complications of this operation were observed in detail, which provided new evidence for the feasibility, safety and effectiveness of Triple P procedure. It is the first time to compare two different methods for uterine preserving procedures and discuss long term prognosis of conservative treatment of PAS with a large sample.

Changes in menstrual and lower extremity discomforts cannot be ignored. Menstrual volumes of 34 pregnant women decreased after surgery, however, there was no significant difference between the observation and control groups. The decrease in menstrual volumes could be associated with extensive invasion of the endometrium by placenta percreta, destruction of the basement layer of endometrium and incomplete repair after operation. It may not be associated with the anterior wall of the uterus which was severely damaged in the resection part of modified “Triple-P” procedure. The decrease in menstrual volume did not harm women without reproductive requirements. Six cases of scar diverticulum were detected by transvaginal ultrasound in 20 women with prolonged menstruation, in which follow-up drugs or surgical treatment was required.

Two patients with successful deliveries after conservative treatment of placenta percreta predicted the possibility of a subsequent pregnancy. However, the risk of recurrent PAS and postpartum hemorrhage should be cautiously evaluated in future. Lower limb complaints were found in quite a few cases. The reason why lower limb complaints were reported by patients with normal echo performance was unclear. We inferred two possible reasons. First, it may be relative to the ischemic injury to the femoral nerve. Second, although vessel radiography and echo examination did not reveal thrombosis in the big artery, it was unclear whether there was small thrombosis in the peripheral artery.

With advances in medical technology, more and more interventional procedures, including temporal balloon occlusion of internal iliac artery (29), common iliac artery (30, 31) or abdominal aorta artery (23, 24) and embolization of uterine artery or internal iliac artery are being developed and are being applied in obstetric diseases. The advantages of these procedures have been elucidated; however, their disadvantages have not been clearly established. Complications of interventional radiological procedures, including thrombus formation, pseudoaneurysm, and arterial rupture among others have been reported. However, most of these complications have been detailed in case reports and long-term outcomes have rarely been recorded or reported (31–34). We concluded that even if imaging examination of the vessels of pelvis and lower extremities returns absolutely normal results, after interventional surgery, patients can still present uncomfortable symptoms for a long period of time. Hematoma and thrombosis are not rare, as reported in previous studies. The causes of thrombosis include hypercoagulability during pregnancy, postoperative compression, puncture injury and slow flow of femoral artery during operation. Blood supply can be temporarily restored by interval deflation, alleviating ischemia and hypoxia of lower abdomen and lower limbs. It may reduce the risk of thrombosis by improving the slow flow of femoral artery. Although we did not observe any significant recent complications of aortic artery, long-term follow-up is needed whether placement of the balloon and method of intraoperative occlusion will cause potential intimal damage and increase the risk of long-term abdominal aortic aneurysm. Therefore, indications of interventional measures should be carefully considered.

## Conclusion

In summary, when combined with tourniquet and/or prophylactic abdominal aorta balloon occlusion, modified Triple-P procedure may be effective in reducing intraoperative blood loss and hysterectomy in patients with placenta percreta. It is a safe and effective surgical alternative to peripartum hysterectomy. However, the complications associated with interventional radiology services should be evaluated furthermore.

## Data Availability Statement

The original contributions presented in the study are included in the article/Supplementary Materials, further inquiries can be directed to the corresponding author/s.

## Ethics Statement

The studies involving human participants were reviewed and approved by Ethics Committee of the First Affiliated Hospital of Zhengzhou University. Written informed consent for participation was not required for this study in accordance with the national legislation and the institutional requirements.

## Author Contributions

HZ and XZ: data collection, manuscript writing, and operation performed. CC: data analysis. YT: figure editing. RG: project development and manuscript editing. All authors contributed to the article and approved the submitted version.

## Conflict of Interest

The authors declare that the research was conducted in the absence of any commercial or financial relationships that could be construed as a potential conflict of interest.

## Publisher's Note

All claims expressed in this article are solely those of the authors and do not necessarily represent those of their affiliated organizations, or those of the publisher, the editors and the reviewers. Any product that may be evaluated in this article, or claim that may be made by its manufacturer, is not guaranteed or endorsed by the publisher.

## Supplementary Material

Supplementary Video 1 Process of the modified Triple P Procedure.

Supplementary Table 1 Raw Data of Effects and long-term outcomes of a modified Triple-P procedure.

## Abbreviations

PAS: Placenta accreta spectrum
EBL: Estimated blood loss
DIC: Disseminated intravascular coagulation
MOF: Multi-organ failure
PPH: Postpartum hemorrhage
MRI: Magnetic Resonance Imaging
DSA: Digital subtraction angiography
ICU: Intensive care unit.
